# 1234. Three Year, Statewide, Assessment of Impact of Time to Sepsis Bundle Completion on Mortality in Patients with Severe Sepsis and Septic Shock

**DOI:** 10.1093/ofid/ofad500.1074

**Published:** 2023-11-27

**Authors:** Joseph Carreno, Alexa Nero, Mckayla Weber, Michael Racz

**Affiliations:** Albany College of Pharmacy and Health Sciences, Albany, New York; Albany College of Pharmacy and Health Sciences, Albany, New York; Albany College of Pharmacy and Health Sciences, Albany, New York; Albany College of Pharmacy and Health Sciences, Albany, New York

## Abstract

**Background:**

In 2013 New York State regulations mandated that all hospitals implement methods for rapidly identifying and treating patients with severe sepsis and septic shock. This study evaluates the impact of those regulations on patients with sepsis between 2016 and 2019.

**Methods:**

Retrospective, statewide, cohort study of all patients in New York State Sepsis Clinical Dataset with severe sepsis or septic shock between 2016 and 2019. Patients were included if they were admitted through the emergency department (ED), had their 3-hour sepsis bundle (blood culture taken prior to antibiotics, antibiotics given within 1 hour, lactate level taken) started within 6 hours of presentation to the ED, and had their sepsis bundle completed within 12 hours of bundle initiation. The main exposure of interest was time to bundle completion from triage in ED. The main outcome of interest was all-cause inpatient mortality. Multilevel logistic regression was used to account for intra-hospital clustering. P-values < 0.05 were considered statistically significant.

Sepsis Clinical Data Disclaimer

**Figure:**
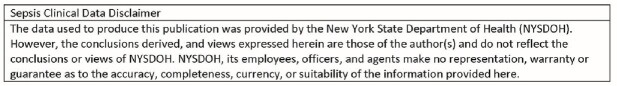

The data used to produce this publication was provided by the New York State Department of Health (NYSDOH). However, the conclusions derived, and views expressed herein are those of the author(s) and do not reflect the conclusions or views of NYSDOH. NYSDOH, its employees, officers, and agents make no representation, warranty or guarantee as to the accuracy, completeness, currency, or suitability of the information provided here.

**Results:**

The weighted sample included 64,119 patients. Baseline characteristics are presented in Table 1. Most individuals (73.6%) had their bundles completed within 3-hours. The median (IQR) time to bundle completion was 1.83 h (1.07 – 3.12), the median (IQR) time to antibiotics was 1.72 h (1.00 – 2.83). Bundle completion within 3-hours from triage was associated with decreased risk of mortality in univariable analysis (17.07% vs. 18.59%, ≤ 3 hrs vs. > 3 hrs, respectively, p < 0.0001). In the multivariable analysis, delayed bundle completion ( >3 h from triage) was associated with increased adjusted odds ratio (aOR, 95% CI) for mortality (1.097 [1.041 – 1.155], P < 0.0001). Delayed antibiotics administration ( > 3 h from triage) was associated increased odds of mortality (aOR [95% CI]: 1.089 [1.031 - 1.151], P < 0.0001). Subgroup analyses are presented in Figure 1 and 2.

**Figure:**
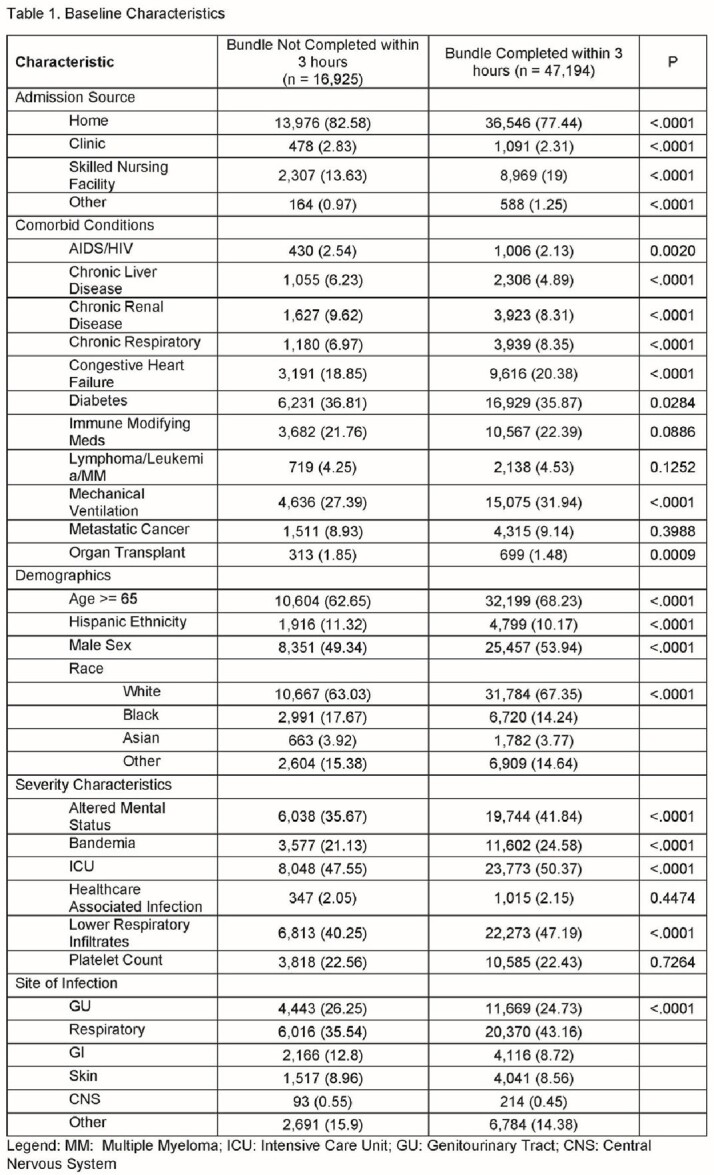

**Figure 2.** Impact of Delayed Antibiotics on Mortality in Subgroups

**Figure:**
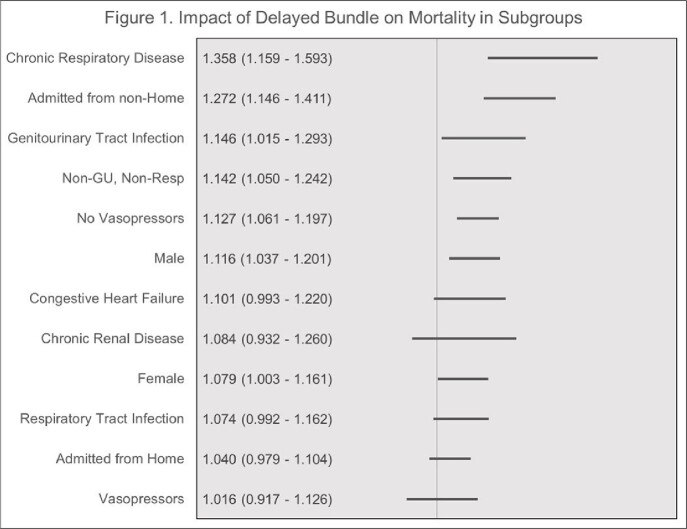

Data are for adjusted odds ratios and indicate adjusted odds ratios for > 3 delay on mortality within subgroup.

**Conclusion:**

Rapid (≤ 3h) completion of sepsis bundle was associated with decreased risk of mortality. This association was also observed for rapid administration of antibiotics. These data suggest that statewide mandates may improve outcomes for patients hospitalized with sepsis.

**Figure:**
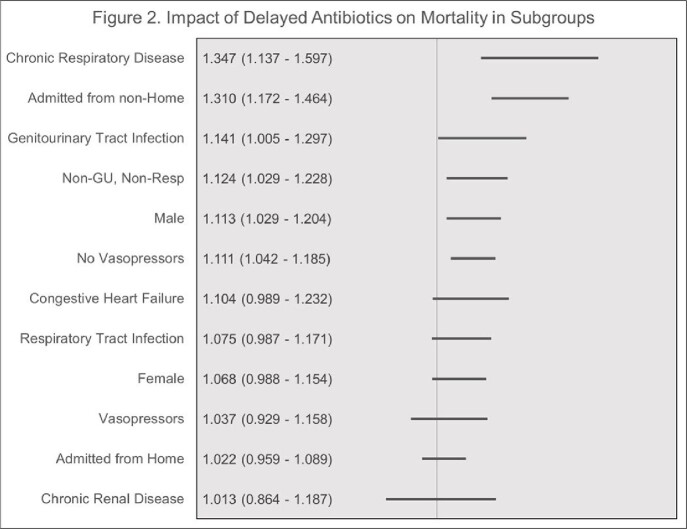

**Disclosures:**

**All Authors**: No reported disclosures

